# Identification of a Novel Class of Farnesylation Targets by Structure-Based Modeling of Binding Specificity

**DOI:** 10.1371/journal.pcbi.1002170

**Published:** 2011-10-06

**Authors:** Nir London, Corissa L. Lamphear, James L. Hougland, Carol A. Fierke, Ora Schueler-Furman

**Affiliations:** 1 Department of Microbiology and Molecular Genetics, Institute for Medical Research Israel-Canada, Hadassah Medical School, The Hebrew University, Jerusalem, Israel; 2 Department of Biological Chemistry, University of Michigan, Ann Arbor, Michigan, United States of America; 3 Department of Chemistry, University of Michigan, Ann Arbor, Michigan, United States of America; Massachusetts Institute of Technology, United States of America

## Abstract

Farnesylation is an important post-translational modification catalyzed by farnesyltransferase (FTase). Until recently it was believed that a C-terminal CaaX motif is required for farnesylation, but recent experiments have revealed larger substrate diversity. In this study, we propose a general structural modeling scheme to account for peptide binding specificity and recapitulate the experimentally derived selectivity profile of FTase *in vitro*. In addition to highly accurate recovery of known FTase targets, we also identify a range of novel potential targets in the human genome, including a new substrate class with an acidic C-terminal residue (CxxD/E). *In vitro* experiments verified farnesylation of 26/29 tested peptides, including both novel human targets, as well as peptides predicted to tightly bind FTase. This study extends the putative range of biological farnesylation substrates. Moreover, it suggests that the ability of a peptide to bind FTase is a main determinant for the farnesylation reaction. Finally, simple adaptation of our approach can contribute to more accurate and complete elucidation of peptide-mediated interactions and modifications in the cell.

## Author Summary

Linear sequence motifs serve as recognition sites for protein-protein interactions as well as for post-translational modifications. One such motif is the CaaX box located at protein C-termini that serves as prenylation site. This prenylation is critical for many signal transduction related proteins and it is thus an important goal to uncover the range of prenylated proteins. Due to poor generalization ability, sequence based computational methods can only go so far in predicting novel targets. In this study, we introduce a novel structure based modeling approach that allows both recovery of known farnesylation substrates, as well as detection of a new class of farnesylation targets. We demonstrate high accuracy in retrospective discrimination between substrates and non-substrates of farnesyltransferase (FTase). More importantly, in a prospective study, *in vitro* experiments validate that 26/29 predicted peptides indeed undergo farnesylation. These novel peptides were derived either from actual human proteins, or predicted to bind particularly well to FTase. Other than the discovery of putative novel farnesylation targets in the human genome, as well as possible inhibitors, we provide insights into the main determinants of farnesylation. Our approach could be easily extended to additional peptide-protein interactions and help the elucidation of the cellular peptide-protein interaction network.

## Introduction

Protein prenylation is a post-translational modification in which a prenyl group (farnesyl or geranylgeranyl) is attached to the protein via a thioether bond to a cysteine at or near the carboxy terminus of the protein (reviewed in [1], [2]). Protein farnesyltransferase (FTase) and geranylgeranyltransferase type I (GGTase-I) are also called CaaX prenyltransferases, due to their ability to catalyze modification of peptides and substrate proteins bearing the carboxy terminal (C’) Cys-aliphatic-aliphatic-variable amino acid (Ca_1_a_2_X) motif [3].

Upon binding of the substrate and the C-terminal Ca_1_a_2_X motif, the catalytic zinc ion of FTase coordinates the thiol side chain of the cysteine and catalyzes the covalent attachment of the lipid anchor to this residue. A detailed view of this mechanism has been obtained by a series of structures solved at different stages of the reaction [4]. After the covalent attachment of the isoprenoid in the cytoplasm, substrate proteins can undergo further processing, resulting in a C’ structure that is able to serve as a specific recognition motif in certain protein-protein interactions [5] and to direct the modified protein towards incorporation into cellular membranes [6].

A wide range of proteins involved in diverse cellular functions require this post-translational modification for their action [2]. While numerous proteins have been experimentally shown to undergo farnesylation *in vivo* [7], [8], [9], it is likely that many FTase substrates remain to be discovered. There is a wide interest in the mapping of FTase targets in the genome, in part due to the therapeutic potential of FTase inhibitors against cancer [10], [11], [12], as well as parasitic infection [13], [14]. Identification of new targets might lead to novel therapeutic approaches [15]. Moreover, the elucidation of cellular FTase targets might shed light on the function of various proteins, as well as on the cellular network of interactions.

Computational approaches have predicted FTase targets based on sequence features of known targets [7], [8]. These methods show good performance in terms of sensitivity, *i.e.* known targets are correctly identified. Thus, prenylation is mainly defined by the last four residues of the protein, although additional weaker sequence constraints have also been identified upstream in the sequence [16]. Other approaches were based on manual inspection and derived from structural features [9].

Substrate specificity has also been examined using peptide libraries. A comprehensive study by Hougland *et al.* on the farnesylation of a large synthetic peptide library has allowed a detailed characterization of FTase specificity [17]. In addition to compiling a large and clean dataset of peptides that contains both efficient substrates and non-substrates for FTase, this study discovered a third group of sequences that are farnesylated only under single-turnover (STO) conditions ([E]>[S]). Analysis of peptide substrates has also demonstrated that reactivity depends on synergy between the side chains at the a_2_ and X positions [18]. These findings indicate that FTase substrate recognition is more complex than the simple Ca_1_a_2_X motif model, and that non-canonical sequences can serve as substrates.

**Figure 1:**
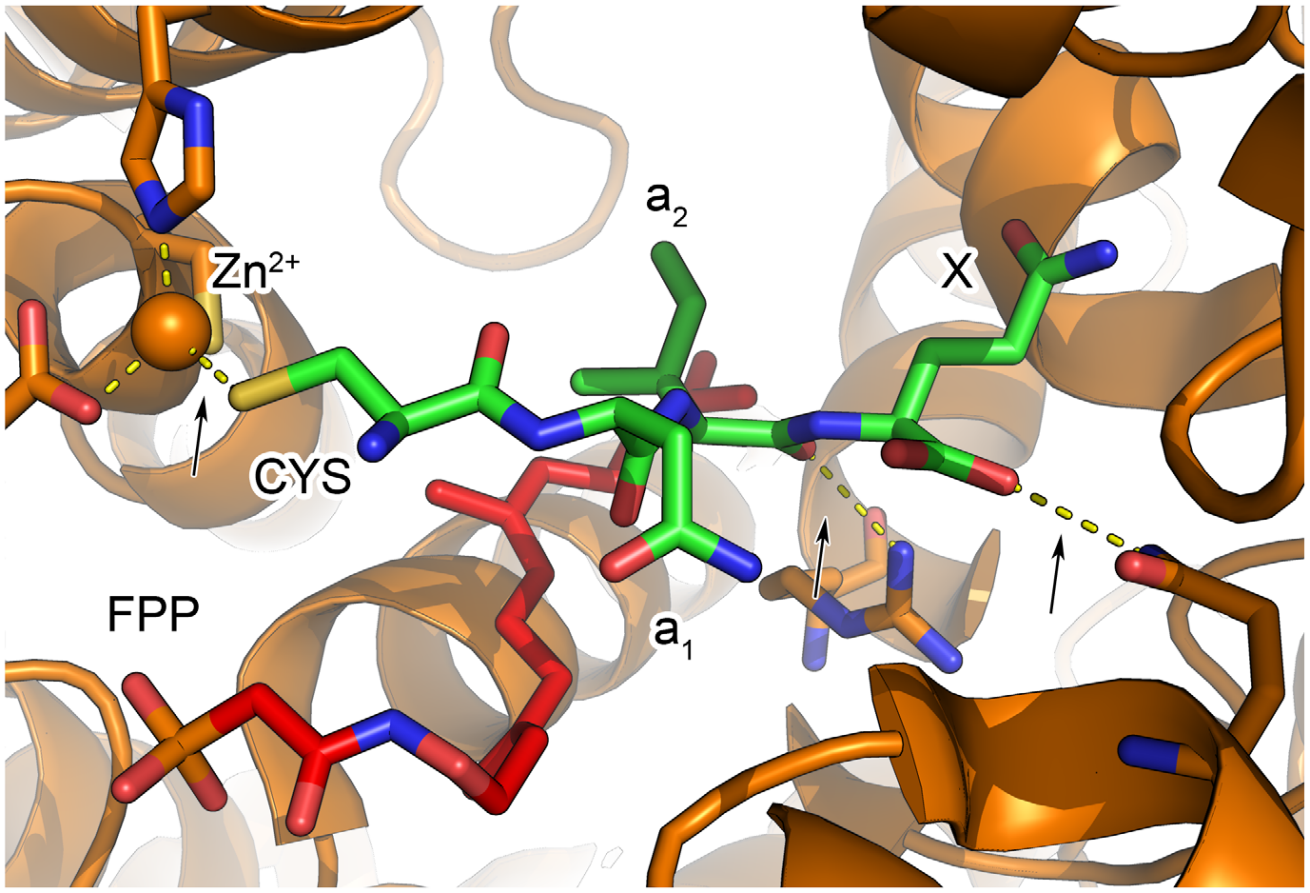
Structural overview of the FTase binding pocket. A top view of the binding pocket of human FTase (orange) in complex with C’ CNIQ peptide in Rap2a (green), and a farnesyl analog (red) (PDB: 1tn6 [9]). Arrows indicate the constraints used during the simulations: the two structurally conserved hydrogen bonds (C’ carboxylate to FTase Q167α and the a_2_ backbone carbonyl oxygen to FTase R202β), as well as the sulfur-Zn^2+^ coordination. The figure was created using PyMOL (http://www.pymol.org).

A large number of structures have been determined for FTase and FTase-substrate peptide complexes [19]. The peptide binding pocket is well-characterized, although a structure of the ternary FTase•farnesyl diphosphate(FPP)•peptide in an active conformation has not been determined [9]. The Ca_1_a_2_X cysteine sulfur atom (prior to the product formation) coordinates the catalytic Zn^2+^ ion together with side chains (D297, C299 and H362) of the FTase β-subunit. The a_1_ side chain points out of the binding pocket and faces the solvent, while the a_2_ side chain is buried within the binding pocket and interacts both with the farnesyl chain of FPP and the residues lining the pocket. The C’ X position interacts with residues mostly from the FTase β-subunit and is considered the main determinant for the specificity between FTase and GGTase-I 9. Finally, two highly conserved hydrogen bonds are formed: 1) between the C-terminal carboxylate group and the side chain of FTase Q167α and 2) between the a_2_ backbone carbonyl oxygen and the side chain of FTase R202β (Figure 1). Despite this detailed structural information, only a handful of different peptide sequences have been solved in complex with FTase.

We previously developed a scheme for modeling the structures of peptide-protein complexes (Rosetta FlexPepDock [20]), which is incorporated within the Rosetta modeling suite framework [21]. This protocol is the starting point for the development of a structure-based scheme for the prediction of peptide binding specificity (FlexPepBind). Specifically, to refine FlexPepBind for the prediction of FTase binding peptides, we have incorporated constraints derived from the conserved features in solved FTase structures and adapted the energy function to distinguish between reacting and non-reacting tetrapeptides (based on an underlying assumption that tetrapeptides that bind will react, while those that do not bind will not react). We trained and tested this protocol against the recent dataset published by Hougland *et al.* [17]. Validation of the protocol against several independent sets showed accurate prediction of peptides that could be farnesylated, both under multiple turnover (MTO) and single turnover (STO) conditions. Evaluation of all possible Cxxx peptides identified a previously uncharacterized class of farnesylation targets that contain an acidic C-terminal residue. The 13 peptides predicted to bind with best affinity were experimentally shown to indeed undergo farnesylation *in vitro*. Finally, a genomic scan for novel FTase targets revealed 77 novel putative FTase targets previously undetected by sequence-based approaches. Among these, 13 out of 16 selected novel putative farnesylation targets were indeed farnesylated by FTase in an *in vitro* experimental validation. FTase-peptide binding is a model system for our approach to peptide-protein binding specificity prediction and design. Our protocol can easily be adapted to additional peptide-protein interactions where both experimental structure and affinity data are available, thereby providing a mechanism to identify targets not detectable by sequence conservation only.

## Results

### FlexPepBind discrimination of FTase binding and non-binding peptides

Recently Hougland *et al.* performed a large-scale study, in which they characterized a TKCxxx peptide library for reactivity with rat protein farnesyltransferase (rat FTase) [17]. Out of an initial library of 213 sequences, 77 peptides are farnesylated under multiple turnover (MTO) conditions, and 51 sequences are not farnesylated under any conditions. Interestingly, the remaining 85 sequences are farnesylated under single turnover (STO) conditions but not under MTO conditions.

We set out to use FlexPepBind and the structural data available for FTase to discriminate MTO sequences from non-reactive (NON) peptide sequences, using the 77 MTO and 51 NON peptide sequences as our training set (128 peptides in total; Dataset S1A). Towards this end, we used the high resolution structure of human FTase in complex with a peptide derived from the carboxy terminus of Rap2a and a farnesyl diphosphate (FPP) analog (PDB: 1tn6 [9]) to create a starting model. The bound peptide was truncated to include only the terminal Ca_1_a_2_X motif. Different peptide sequences were then threaded onto the peptide backbone and used as starting structures.

Initially, we modeled peptide-FTase complex structures for different peptide sequences by applying the Rosetta FlexPepDock protocol to the threaded starting models. This protocol was developed previously in our lab for the modeling and refinement of peptide-protein complex structures to high resolution [20]. Our simulations included three constraints, namely the conservation of the 2 structurally conserved hydrogen bonds (C’ carboxylate - FTase Q167α; a_2_ backbone carbonyl oxygen - FTase R202β) and the location of the cysteine sulfur atom coordinating the Zn^2+^ ion (Figure 1, see Methods for more details).

**Figure 2:**
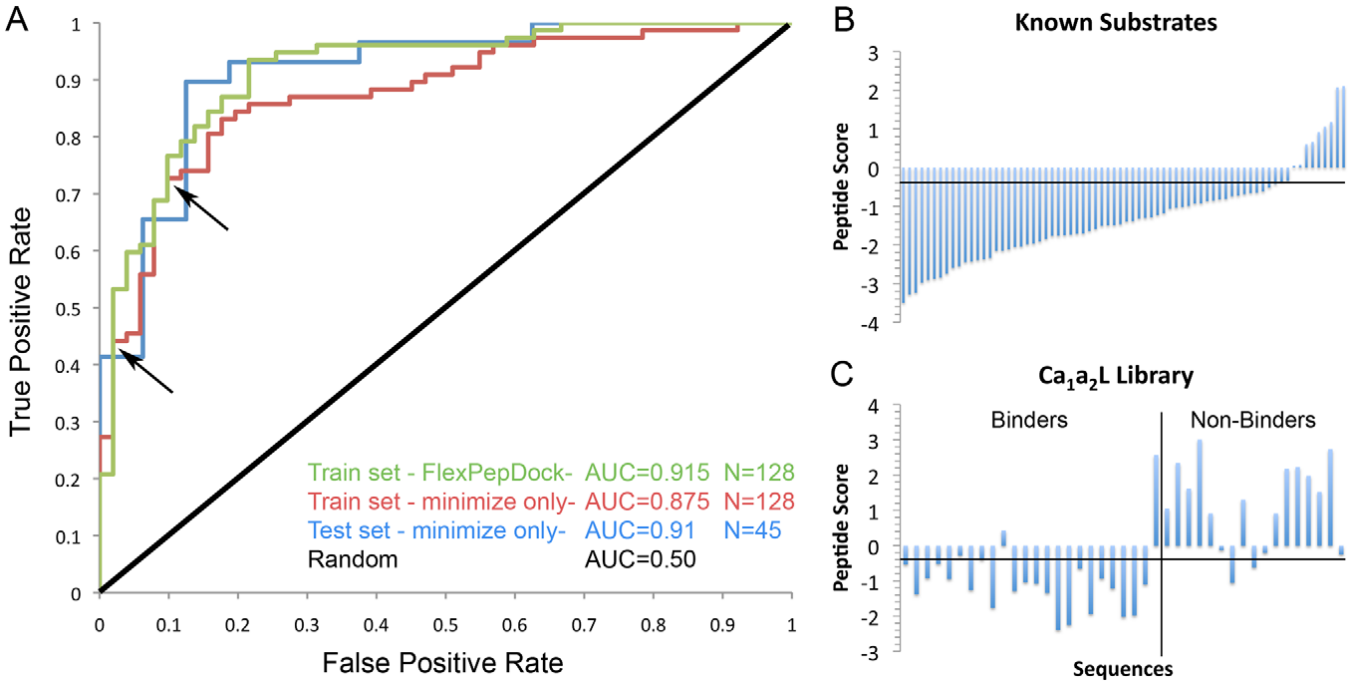
FlexPepBind allows good discrimination between substrate - and non-substrate sequences. **A.** ROC-plot of the discrimination between MTO peptide sequences and non-active peptide sequences on the training set with the FlexPepDock based protocol (green), the fast, minimization based protocol (red), an independent test set (blue), and expected random discrimination (black). The Area Under the ROC Curve (AUC) value for the training set is 0.915/0.875 for the FlexPepDock and minimization based protocols, accordingly. Note that the performance of the minimization-based protocol on the test set is even better than on the training set (0.91 vs. 0.875). For the indicated points on the plot, an energy threshold of -0.4 corresponds to a 69% True Positive Rate (TPR) and 8% False Positive Rate (FPR). A more stringent threshold of -1.1 energy units corresponds to a 44% TPR and 2% FPR. Training and test sets are detailed in Dataset S1A&B. **B+C. Validation on additional independent test sets shows robust and reliable performance of our modeling protocol.** **B.** The distribution of energies for known FTase substrate sequences. The horizontal line indicates the -0.4 threshold obtained from the training set (see Text). Using this criterion, 85% of the known binders are recovered. Note that this corresponds to a significantly better TPR than the one obtained on the training set. **C.** Energy distribution for a synthetic library of Ca_1_a_2_L peptides investigated in Krzysiak *et al.* [23]. As in B., the horizontal line indicates a threshold of -0.4, which in this case displays 87.5% TP and 12.5% FP rates (*i.e.,* only 3 false negatives and 2 false positives). The peptide sequences and scores can be found in Dataset S1C&D.

For each simulation, the energy of the best scoring Cxxx peptide was extracted (see Methods for further details). Figure 2A shows the Receiver Operating Characteristic (ROC) plot for the ability of the peptide energy to discriminate between MTO sequences and non-substrate sequences. The plot shows very good discrimination with an Area Under the ROC Curve (AUC) value of 0.915 on our training set.

These results demonstrate that a structure-based evaluation of the peptide energy can distinguish very well between farnesylated and non-farnesylated peptide sequences. Since the known constraints restrict the simulation to a closely defined region in the binding site, we reasoned that a simpler and faster protocol might be able to model the peptides with similar accuracy. Our simplified protocol therefore includes only a minimization using the Rosetta energy function [21], [22] under constraints to retain the 2 structurally conserved hydrogen bonds and the cysteine sulfur atom location coordinating the Zn^2+^ ion (see above and Methods for more details). This protocol yielded similar results with an AUC value of 0.875 on the training set. A peptide energy threshold of -0.4 (*i.e.* sequences with energy below/above -0.4 are predicted to be binders/non-binders and therefore farnesylated/non-farnesylated, respectively) corresponds to a 69% True Positive Rate (TPR) and 8% False Positive Rate (FPR). A more stringent threshold of -1.1 energy units corresponds to a 44% TPR and 2% FPR (Figure 2A). With the two protocols exhibiting similar performance, we decided to proceed further using the fast minimization protocol. (Performance on the training set using additional sampling and scoring schemes is summarized in Table S1.)

### Validation of FlexPepBind on independent test sets

To assess FlexPepBind using the selected thresholds, we evaluated performance on three independent test sets (Dataset S1B-D online).

#### 1. Secondary synthetic library (Dataset S1B)

In their original paper, Hougland *et al.* [17] assayed the activity of a secondary synthetic peptide library, biased towards sequences containing canonical amino acids at the a_2_ and X positions. In this library, 29 peptides displayed MTO activity with FTase and 15 peptides were not reactive. The sequences from this library were not used at any stage during the development of our protocol. The ROC plot for this test set in Figure 2A shows an AUC value of 0.913 that is even better than for the training set. Applying the thresholds identified in the training set yields 86% TPR/12.5% FPR for the −0.4 threshold, and 72%/12.5% for the −1.1 threshold, respectively.

#### 2. Known FTase substrate sequences (Dataset S1C)

This dataset is based on Table S1 from the study by Hougland *et al.* [17] which lists the carboxy terminal sequences of known proteins that serve as substrates for FTase, collected from different studies [7], [8], [9]. Figure 2B shows the energy distribution of the known sequences, as estimated by FlexPepBind. Applying the thresholds obtained from the training set, we are able to recover 64% of the known substrates with the stringent threshold, and 85% of the known substrates with the less restrictive criterion. These values are much better than the TPR obtained for the training set.

#### 3. Ca_1_a_2_L library (Dataset S1D)

In a recent work by Krzysiak *et al.* [23], a synthetic library of peptides of the form Ca_1_a_2_L, “canonical” GGTase-I substrates, was characterized for reactivity with FTase. In this study, sequences for which product conversion was detected by HPLC were labeled as ‘true’ substrates, while sequences for which no conversion was detected were labeled as ‘false’ substrates [23]. Using the threshold of -0.4 results in predictions with 87.5% TPR and 17.5% FPR, consistent with the performance on other peptide libraries (Figure 2C). These results demonstrate that the C' residue is not necessarily the main determinant of FTase substrate selectivity.

### Exploration of the full substrate sequence space

**Figure 3:**
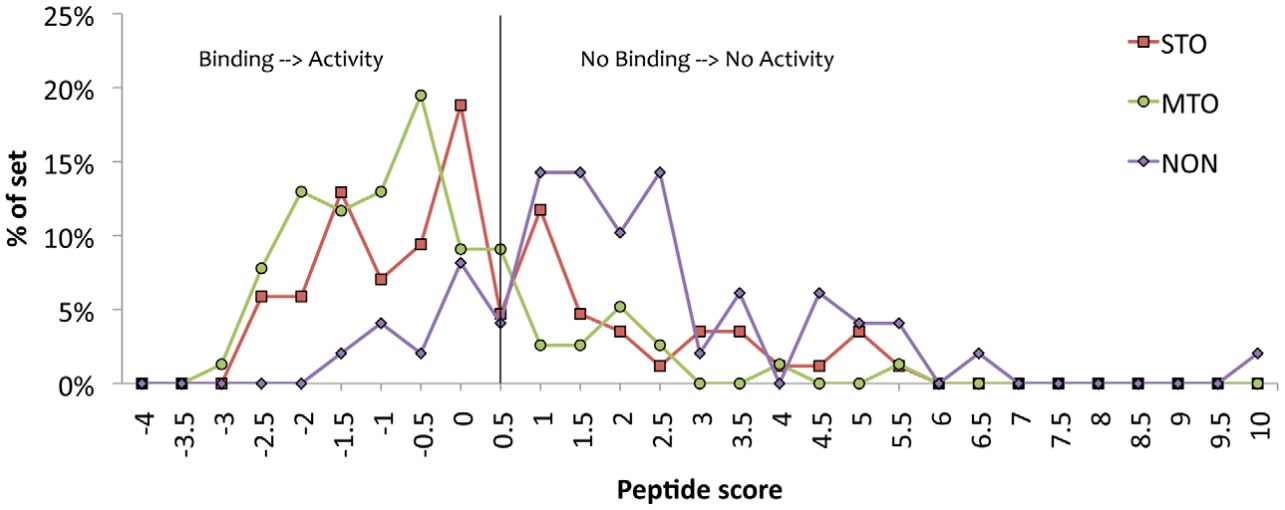
Energy distribution of all possible Cxxx sequences, as well as previously characterized peptides (STO, MTO and NON) [**17**]. The distributions of known single turnover (STO) and multiple turnover (MTO) peptide sequences overlap, and are both significantly shifted towards low peptide energies, compared to peptide sequences that do not undergo farnesylation (NON). The thresholds obtained for the discrimination of MTO/NON predict 1349 (17%; -1.1 threshold) and 2309 (29%; -0.4 threshold) of the possible tetramer peptide sequences to undergo farnesylation.

**Figure 4:**
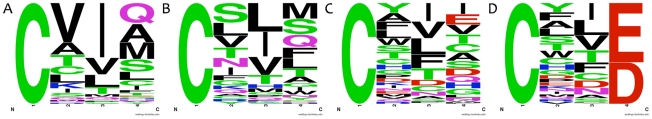
A novel class of farnesylation targets. The sequence logos of different sets of Farnesylation targets are shown for **A.** 72 known substrates (Dataset S1C); **B.** 77 MTO peptides from Dataset S1A; **C.** 1349 (out of 8000) sequences that pass the stringent threshold of -1.1 and are predicted to undergo farnesylation – while position a_2_ of the motif is still prominently aliphatic (ILE/VAL/LEU/PHE), positions a_1_ and X are much more versatile than expected; **D.** A subset of **C** with D/E at C-terminal position (238/1349) constitutes a novel substrate class for FTase (Logos created by http://weblogo.berkeley.edu/).

Using FlexPepBind, we modeled all of the 8000 possible Cxxx sequences and scored them according to our protocol. The thresholds for the discrimination of MTO/NON predict that 1349 (17%; stringent threshold = −1.1) and 2309 (29%; threshold = −0.4) of all tetramer peptide sequences could be possible substrates (see Figure 3). This set of putative farnesylation targets suggest a much more versatile binding motif than previously accepted (see Figure 4): while position a_2_ of the Ca_1_a_2_X motif is still prominently aliphatic (ILE/VAL/LEU/PHE), positions a_1_ and X are less restricted than previously reported (compare Figure 4C to Figures 4A&B). In particular, we identify within this set a novel class of farnesylation targets that contain an acidic residue at the C-terminus (238/1349 putative targets; ∼20%; see Figure 4D).

Figure 4C indicates that the minimization-based protocol tends to miss larger residues at the C-terminal X position. Indeed, assessment of the prediction accuracy for this position on the training set shows that only 1/8 CxxF and 0/3 CxxW sequences are correctly predicted with the chosen protocol (CxxM peptides are predicted with higher accuracy: 10/14). Using the FlexPepDock based protocol, performance increases to: 6/8 CxxF; 2/3 CxxW and 11/14 CxxM, demonstrating that CxxF peptides are indeed rescued by the additional backbone flexibility. Therefore, it might be advisable to use the FlexPepDock based protocol for peptides that contain a bulky C-terminal side chain.

### Comparison to sequence-based approaches

We compared our predictions to the PrePS [7] prediction of prenylation targets on the initial training set of peptides. Regarding the discrimination of MTO substrates from non-active peptides, PrePS results are comparable to FlexPepBind (AUC of 0.92, with a threshold corresponding to 60% TPR for 2% FPR). However, the performance for STO peptides is significantly better for our structure-based approach: while FlexPepBind recovers 47% and 32% of the STOs with the loose and stringent thresholds concordantly, PrePS predicts only 14% of these sequences as substrates.

### Experimental confirmation of novel substrate class

Since our retrospective studies indicated that our approach can very accurately retrieve actual farnesylation targets, we were interested in testing it prospectively – could novel targets be indeed identified? We selected the 13 best scoring peptides (*i.e.* predicted tightest binders), yet previously uncharacterized for experimental validation. These are mostly ‘non-canonical’ peptides, including 5 peptides with an acidic C-terminal residue. Indeed, PrePS [7] predicts only 2 out of the top-scorers to be FTase substrates. *In vitro* farnesylation assays indicate that all of these peptides indeed undergo farnesylation catalyzed by FTase: 10 under MTO conditions and 3 under STO conditions (Table 1A). These results demonstrate the robustness of our protocol and its exceptional accuracy. Importantly, they confirm the novel class of farnesylation substrates that contain a negatively charged C-terminal residue (Figure 4D).

**Table 1:** Experimental evaluation of farnesylation of predicted peptide substrates: 26/29 (90%) of the predictions are indeed farnesylated, including a novel class of farnesylation targets identified in this study.

		PrePS prediction				
Motif	Derived from protein^c^	Full^d^	x-CVLS^e^	H-Ras-Cxxx^f^	Score^a^	Exp. Result^b^
**(A) Top-scoring peptides**						
CYLI				-	-3.96	MTO
**CYLE**				**-**	**-3.82**	**STO**
CYLV				-	-3.60	MTO
CFLV				-	-3.60	MTO
CLII				++	-3.51	MTO
**CYVE**				**-**	**-3.43**	**MTO**
**CYIE**				**-**	**-3.40**	**MTO**
**CFIE**				**-**	**-3.34**	**STO**
CLIV				++	-3.33	MTO
CYLL				-	-3.24	MTO
**CYLD**				**-**	**-3.13**	**MTO**
CWVI				-	-3.03	STO
CWLV				-	-3.01	MTO
**(B) Top-scoring peptides that occur at C-termini of human proteins**						
CYVA	Q9NTW7-3	-	-	+	-2.88	MTO
CFLT	Q2UVF0	--	-	+	-2.74	MTO
CAFI	Q7Z2H8	--	+	-	-2.62	STO
CWLS	A6QL63-3	-	+	+	-2.46	MTO
CCLS	Q9NZM3-3	--	--	++	-2.37	MTO
**CTTE**	**Q5T2R2-2**	**--**	**-**	**-**	**-2.14**	**STO**
CHFH	Q8TCU3-2	---	+	--	-2.14	STO
CKLA	Q9BPZ7-6	-	-	+	-2.06	MTO
CWTC	Q8NFG4-3	-	++	-	-1.94	MTO
CSLI	Q14CB8-5	-	+	++	-1.90	MTO
**CLFE**	**Q9UHP7-3**	**--**	**+**	**--**	**-1.77**	**None**
CPFF	Q8N693	---	-	--	-1.69	STO
CGVG	A6NHS1	-	-	+	-1.65	MTO
CFDI	Q8NEB5	--	++	--	-1.59	None
CHCI	Q99988	--	+	-	-1.56	None
CVCV	O75391	-	+	+	-1.12	MTO

**(A**) Top-scoring peptides. **(B)** Top-scoring peptides that occur at C-termini of human proteins.

The novel class of farnesylation targets identified in this study that contains acidic C-terminal residues (see Figure 4D) are shown in bold.

a Peptide score for sequences as measured by the FlexPepBind protocol developed in this study.

b Experimental validation of farnesylation of predicted peptides in this study (see Methods).

c Uniprot [24] identifier of human proteins containing putative farnesylation motif.

d-f PrePS predictions [7]:

d based on 30 C-terminal residues of protein sequence;

e based on 30 C-terminal residues of protein, with the last 4 residues replaced by the H-Ras canonical Cxxx motif (CVLS) (this indicates the amenability of the upstream sequence to allow farnesylation of the C-terminus);

f based on 30 C-terminal residues of known substrate H-Ras, with last 4 residues replaced by Cxxx motif (this indicates the amenability of the given Cxxx C-terminal sequence to undergo farnesylation within the context of a known strong farnesylation target).

Structural investigation of this novel class of substrates suggests that the negatively charged C' side-chain is stabilized by FTase residue His 149βwhile accepting a hydrogen bond from Trp102β (GLU) and creating an additional hydrogen bond with the side-chain of Ser99β (GLU & ASP) (see Figure S1). Additional polar interactions with water molecules are possible but were not explicitly modeled.

### Genomic scan for novel human FTase targets

Equipped with a score that can predict both known and novel FTase targets, we set out to scan the human genome for proteins that may undergo farnesylation. Our protocol was developed based on experimental assays on rat FTase (and the structure of human FTase [9]). Since rat and human FTases show very high sequence identity (92% and 96% for subunits α and β respectively), and none of the sequence differences are located at or near the peptide binding site, we are confident that our prediction scheme can be applied to human farnesylation as well.

We identified 756 unique proteins in human SwissProt [24] that contain the Cxxx motif at their carboxy terminus. 167 and 309 of these protein sequences obtained scores lower than the −1.1 and −0.4 threshold values, respectively, indicating that these proteins might be farnesylated by FTase. We focused on the group of 167 proteins detected with the more stringent threshold.

Could these proteins indeed be FTase substrates? Several indications support our predictions: First, amongst the 167 candidates, 42 contain a Cxxx motif of a known FTase substrate. Secondly, the Gene Ontology (GO) [25] cellular compartment annotation for most of these 167 proteins is *Membrane related* (see Figure S2; see Methods for more details). This supports their association with membranes, possibly by farnesylation (albeit this localization annotation might have been inferred from sequence similarity). Furthermore, peptide library studies have demonstrated FTase-catalyzed farnesylation (under STO or MTO conditions) of 50 of these Cxxx motifs (representing 66 human proteins) [17]. Finally, analysis of the putative target proteins with the PrePS server predicts that most of them (90/167) are indeed FTase targets, while the other 77 are not predicted to be farnesylated (see Figure S3). To further characterize the latter, we proceeded with *in vitro* experimental validation of selected sequences.

### Experimental validation of human targets

Among these 77 proteins (containing 72 unique Cxxx motifs), 39 motifs had not yet been tested for *in vitro* farnesylation. The second set chosen for experimental validation consisted of 16 top-scoring peptides selected from these 39 motifs. Of the 16 tested peptides, 9 and 4 peptides are farnesylated *in vitro* under MTO and STO conditions, respectively, while only 3 were not farnesylated by FTase (Table 1B). None of the 16 sequences in this second set are predicted to serve as farnesylation targets by PrePS. Interestingly, for 9 of these 16 sequences, PrePS predicts that the upstream context of the motif is suitable for farnesylation. In these cases, the PrePS negative prediction is based on the sequence of the Cxxx motif. This suggests that improved characterization of the contribution of the 4 C-terminal residues to farnesylation can identify more farnesylation targets. Finally, for 8 of these 16 sequences, PrePS would predict farnesylation of the Cxxx motifs in the background of the favorable H-Ras upstream sequence. The balance between the upstream signal and the C-terminal Cxxx motif is therefore an interesting subject for future research.

Most of the proteins identified by this study as novel FTase substrates have not been well characterized to date. Consequently, *in vivo* experiments that evaluate the cellular localization and prenylation status of these proteins, in conjunction with the *in vitro* farnesylation demonstrated in this study, will advance their functional characterization.

## Discussion

We present here a simple and accurate structure-based scheme for prediction of the sequence of FTase-binding peptides. We have validated our protocol against several test sets, and predictions were experimentally verified *in vitro* to reveal novel putative FTase substrates and potential tight binders. This approach has expanded our understanding of farnesylation, both within the context of the reaction itself, as well as in the greater context of cellular biology. Furthermore, this protocol presents an advance in the computational prediction of binding specificity in general.

### Insights into the mechanism of farnesylation from structure-based predictions - Binding affinity vs. reactivity

The protocol that we developed essentially estimates the binding affinity of FTase for Cxxx peptides, using a training set of reactive peptides, rather than predicting the farnesylation activity of these sequences. This has several implications and limitations. Remarkably, the ability to discriminate peptides that undergo MTO reaction from non-active peptides according to binding energy suggests that the non-active peptides may bind weakly or not at all to FTase (see Figure 3). This finding is supported by results from an *in vitro* inhibition experiment in which none of the tested non-active peptides inhibited FTase-catalyzed farnesylation of a known substrate [17]. In turn, the members of the small class of FlexPepBind false positive peptides may bind to FTase with high affinity but still not be farnesylated. These false positive peptides could therefore serve as FTase inhibitors and represent an interesting set to characterize in future work.

Previous studies have shown that the sequence immediately upstream of the conserved cysteine residue may also play a role in substrate selectivity [16]. These sequences modulate peptide affinity and reactivity with FTase, *i.e.* a high-affinity terminal tetramer sequence does not necessarily ensure farnesylation of the protein. For half of the proteins tested in the study, the PrePS [7] program predicts favorable upstream sequences. This result coupled with the high-affinity -Cxxx motif predicted by FlexPepBind (see Results and Table 1B) increases the confidence that the human proteins containing the said Cxxx motif could be farnesylated *in vivo*. In turn, a favorable upstream sequence might compensate for a weak C-terminal signal. Our future work will therefore further characterize the balance between these two signals in determining farnesylation.

### STO peptide substrates

One puzzling aspect of FTase substrate recognition is the large number of peptides that exhibit single turnover activity. The single turnover rate constant, *k*_farn_, reflects all of the rate constants up to but not including the release of the farnesylated product [4], [26], [27], [28]. Therefore, the STO peptides bind to FTase and are readily farnesylated, but the product dissociates very slowly so multiple turnover activity is very slow. Consistent with this, FlexPepBind achieves an AUC value of 0.776 in the discrimination between STO and non-active peptides on the training set, indicating that STO peptides have higher affinity for FTase than the non-active peptides (see Figure 3). Our protocol thus identifies STO peptides much better than sequence-based methods (see Results and Hougland *et al.* [17]).

What then discriminates between MTO and STO peptides? Hougland *et al.* postulated that the farnesylated STO peptides might bind more tightly to FTase than farnesylated MTO peptides, and as a consequence FPP-catalyzed product dissociation is slow [17]. However, binding energy, as approximated by our approach, seems to be a poor discriminator between MTO and STO peptides (AUC value of 0.625 on the training set – Dataset S1B). That is, estimation of the binding affinity of peptides in the context of static conformations of the protein cannot explain the difference in reactivity. Furthermore, application of this approach to models of MTO and STO peptides at different stages of the reaction sequence (pre-farnesylation, post-farnesylation with the farnesyl group in the exit groove) was not able to account for this difference as well. Hence, rather than binding affinity, a parameter related to the *dynamics of product dissociation* might dictate turnover. We therefore conclude that a dynamical approach, such as molecular dynamics, will be required to explain the mechanism that distinguishes STO from MTO peptides.

### Are the proteins corresponding to MTO and STO peptides FTase substrates in vivo?

Past *in vitro* peptide farnesylation experiments with FTase have measured *k*_cat_/*K*_M_^peptide^ under MTO conditions and *k*_farn_ rate constants under STO conditions [17]. The estimated reactivity of MTO and STO peptides (see Methods) measured in this work falls within the range of previously measured activity [17]. Therefore, these peptides have comparable reactivity to other substrates, including peptides that correspond to proteins that are farnesylated *in vivo.*

Measured under MTO conditions, the kinetic parameter *k*_cat_/*K*_M_^peptide^ is termed the specificity constant and best reflects the reactivity of an enzyme in the presence of multiple substrates, as observed *in vivo* [29]. In a cell, the reactivity of a protein substrate with FTase depends on the value of *k*_cat_/*K*_M_^peptide^ as well as on the concentration of the substrate within the cytosol. Although a protein substrate with a higher value of *k*_cat_/*K*_M_^peptide^ is more likely to be farnesylated *in vivo*, it is unclear what level of *in vitro* activity corresponds to a true FTase substrate *in vivo*. Furthermore, *in vivo* the optimal levels of farnesylation of a given substrate may vary and a low fraction of modification may still be biologically relevant. Additionally, a substrate must be localized to the proper cellular locale in order for modification to occur and the C-terminus of the protein must be structurally available. Peptide library studies and this work have aided in determining potential FTase substrates and have also identified already known substrates, but more work is needed to characterize the reactivity of these substrates *in vivo*.

As for the STO-only peptides, these substrates are readily farnesylated but the product does not dissociate rapidly. One possibility is that these proteins function as FTase inhibitors and consequently play a regulatory role within the cell [17]. However, both FPP and peptides have been implicated in catalyzing product dissociation of farnesylated STO peptides [17], [30], [31] and therefore it is possible that other cellular components could activate product dissociation allowing rapid farnesylation of these proteins *in vivo*. Therefore, competition or synergy among different FTase substrates could play an important functional role for modification and localization of proteins. Improved identification of STO peptides using the structure-based FlexPepBind approach presented here will expand our understanding of regulatory aspects of this reaction. In addition, the overlap in substrate preference of FTase and GGTase-I [3] indicates that modulation of the type of prenyl modification (e.g. changes in relative enzyme availability or magnesium concentration) might be functionally important as well. Our future focus on structure-based characterization of GGTase-I specificity will allow an improved investigation of this regulatory feature, complementary to sequence-based studies [7], [8].

### Identification of new putative farnesylation targets

Scanning the human genome for putative FTase targets using our structure-based approach revealed many putative, not yet detected, farnesylated proteins. These new farnesylation substrates may provide novel disease targets for farnesyltransferase inhibitors. Moreover, the prediction that these proteins are farnesylated might shed light on their function. As an example, the putative proteins Q8NA34, A6NHS1, and P0C7P2 (UniProt identifiers [24]) all contain C' sequences strongly predicted to serve as farnesylation targets suggesting that the proteins are membrane localized. Additionally, our method also predicts FTase substrates that have recently been identified from *in vivo* experiments. For example, Kho *et al*. used a tagging-via-substrate proteomic approach to discover novel farnesylation targets [32]. They found a total of 18 farnesylated proteins: 13 are well known, and of the remaining 5 our approach predicts 4 to be farnesylated, including one hypothetical protein. Furthermore, it was recently found that pathogens can hijack the host farnesylation machinery to their own advantage, for example, anchoring effector proteins to the membrane of *Legionella*-containing vacuoles [33], [34], [35]. Thus, in addition to the identification of putative new farnesylation targets in the human genome, FlexPepBind can be used to scan pathogen genomes for farnesylation as well.

### The biological relevance of putative novel targets

13/16 motifs derived from human proteins tested for *in vitro* farnesylation indeed undergo the reaction. Will this also happen *in vivo*? In the following we compile additional available details on these targets that might help answer this question.

One way to assess the *in vivo* relevance of the observed *in vitro* ability to undergo farnesylation of the C-terminus of a protein is to look for homologous proteins that also undergo farnesylation. Such information can easily be retrieved from PRENbase [8]. A search in this database revealed that Kinesin-like protein KIF21B variant (Q2UVF0; CFLT) maps to a cluster of 9 highly similar eukaryotic sequences (E-val<e-20) that are all predicted to undergo farnesylation by PrePS. Similarly, Ankyrin repeat and BTB/POZ domain-containing protein BTBD11 (A6QL63-3; CWLS) maps to a cluster of 25 sequences of related proteins in PRENbase. Zinc finger protein 64 homolog (Q9NTW7-3; CYVA) also contains a number of conserved homologs in PRENbase, however in this specific isoform the target cysteine is part of the Zinc-finger structural motif, and therefore it might not readily be farnesylated.

Another interesting putative farnesylation target that we have identified is the short isoform of Intersectin-2 protein (Q9NZM3-3; CCLS). This protein is involved in clathrin-mediated endocytosis [36], [37], and farnesylation could be a mechanism for regulation and localization to the membrane, similar to the prenylation of Rho GTPases for endocytosis [38]. In particular, the long isoform of intersectin-2 contains additional domains [39], including a PH domain known to bind phosphoinositides [40], and a C2 domain known to be involved in Ca-dependent and independent binding of phospholipids [41]. Consequently, in the short isoform that lacks these domains, farnesylation might indeed be used as an alternative way to achieve membrane proximity and attachment. While the localization of some Rho GAP proteins (e.g. p190 [42]) is regulated by phosphorylation, the short isoform of Rho GTPase-activating protein (GAP) 19 (Q14CB8-5; CSLI) exposes a new C' motif that may target it to the membrane (while keeping the Rho GAP domain intact). The same goes for MAPKAP1 isoform 6 (Q9BPZ7-6; CKLA), a subunit of mTORC2. While the full length protein was shown to contain a functional PH and Ras binding domains [43], the truncated isoform reveals a C' putative farnesylation motif instead. Thus, for all but three MTO sequences we could gather additional information that supports actual *in vivo* farnesylation. We further discuss alternative splicing as a regulatory mechanism below.

Four motifs were found to undergo *in vitro* farnesylation under STO conditions. The Homeobox protein ESX1 (Q8N693; CPFF) is cleaved into an N' and C' domain; while the N' enters the nucleus, the C' domain is localized to the cytoplasm where it inhibits cyclin degradation[44]. A search for homologues in PRENbase produced a cluster with 2 sequences predicted to undergo farnesylation by PrePS. While the latter could support actual farnesylation of this protein, in this case this modification would serve for purposes other than membrane association, such as the interaction with new partners [5]. Isoform 2 of the integral membrane protein solute carrier family 7 member 13 (Q8TCU3-2; CHFH) is missing an intracellular domain, and therefore places its C' in proximity to the membrane. Here farnesylation could play a role in targeting this transmembrane protein to a specific membrane compartment [45], resulting in different membrane distributions for alternative spliced isoforms. Decaprenyl-diphosphate synthase subunit 1 isoform (Q5T2R2-2; CTTE) is a nuclear encoded mitochondrial protein. If indeed farnesylated, this would be a first example where an isoform of a mitochondrial protein is farnesylated in the cytosol. Finally, the proton-coupled amino acid transporter 1 (Q7Z2H8; CAFI) is likely not a farnesylation target, since mutation of the target cysteine to alanine did not affect its function [46]. As discussed above, the biological role of farnesylation under STO conditions is not yet clear; furthermore, if these proteins are farnesylated *in vivo,* the function is likely more complex than localization to the membrane.

For the three motifs that were not farnesylated under *in vitro* conditions, additional information about the cognate proteins indeed suggests that the C-terminal cysteines are likely not farnesylated *in vivo*. The target cysteines of Growth/differentiation factor 15 (Q99988; CHCI) and the extracellular C-type lectin domain family 2 member D isoform (Q9UHP7-3; CLFE) are part of a conserved disulfide bridge and therefore most likely not farnesylated *in vivo.*

In this study, we chose peptide motifs for *in vitro* experimental characterization based on their predicted ability to bind FTase and their novelty (*i.e.* not predicted by PrePS, and not yet experimentally tested). While our *post-hoc* literature analysis reinforces some of the predictions, other targets will apparently undergo farnesylation only *in vitro*. The latter represent an interesting set of proteins that allow the investigation of additional factors that regulate the actual farnesylation *in vivo*, and that therefore distinguish between the ability of a protein to undergo farnesylation *in vitro* and *in vivo*. In any case, future *in vivo* validation is required for all putative targets to unequivocally define their functional importance in the cell.

### Alternative splicing as regulator of farnesylation

Approximately half of the proteins strongly predicted by FlexPepBind to undergo farnesylation (86/167) appear in alternative splicing isoforms (according to Swissprot [24]; the actual number of isoforms is expected to be higher, as more experimental data accumulate from large scale sequencing efforts). Among these 86 proteins, most (61) contain the Cxxx motif only in some of the isoforms. This may present a second layer of regulation for the localization of such proteins, in which a protein can reside in different cellular compartments as a function of the isoform expressed at a given time or a given tissue and therefore perform different functions. This form of regulation may be a consequence of the irreversible nature of farnesylation. On the other hand, farnesylation can be maintained despite alternative splicing. For example, in Rab28 the two reported isoforms (hRab28S, hRab28L) differ only by a 95-bp insertion within the coding region [47]. This insertion generates two alternative sequences in the 30 C-terminal amino acids, which strikingly both contain a high-affinity farnesylation motif (CSVQ – L isoform, CAVQ – S isoform) at the C-terminus. This is similar to the case of KRas that also expresses as two splice variants with strong farnesylation motifs (CIIM - 2A isoform, CVIM - 2B isoform) and different upstream sequences. In this case one upstream sequence harbors an additional palmitoylation site, and may thus lead to different distribution in the membrane [48].

### Computational approaches for the prediction of binding specificity – challenges and successes

FlexPepBind is a framework for designing peptides that bind to a given protein, as well as for the prediction of peptide binding specificity. It is based on our previously developed modeling protocol FlexPepDock for peptide-protein structures [20]. Inclusion of constraints derived from known structures with bound peptides allows for the definition of backbone flexibility that is appropriate for the specific system of interest, and optimization of the energy function is based on a given set of binding and non-binding peptides.

How much conformational freedom should be given to the peptide in order to sample the correct conformation, without introducing too much noise? What is the best score for discrimination of active and non-active peptides? While Grigoryan *et al.* were able to design peptides that bind to specific members of the bZip family [49], Goldschmidt *et al.* identified fibril-forming peptides on a large scale [50], and Kota *et al.* defined a binding motif for type I HSP40 peptide substrates [51] using fixed backbone conformations, the incorporation of backbone conformational flexibility has generally improved computer-aided design of functional protein interactions, as well as structure-based prediction of peptide-protein and protein-protein interaction specificity [52]. In particular, a range of backbone conformations created by the backrub method [53] improved computational sequence recovery of experimental phage display results on human growth hormone [54], and variation along normal modes allowed improved optimization of binding between the anti-apoptotic protein BCL-xl and BH3 helical ligands [55]. Modeling of the structure of HIV protease – peptide targets using a flexible docking protocol allowed the distinction between peptides that are cleaved from those that are not, opening new avenues towards the design of HIV protease inhibitors [56].

In our modeling study of FTase binding peptides, side-chain repacking alone that restricts sampling to a discrete rotameric representation results in a low AUC value of 0.606 over the training set. Simple minimization that allows for very subtle backbone, side chain, and rigid-body adjustments relieves clashes that cannot be resolved with a simple rotameric side-chain search, and indeed improves performance significantly (AUC = 0.875). Much more extensive sampling with Rosetta FlexPepDock [20] produces even better AUC values (up to 0.94). Therefore, the more we sample, the better we perform. On the other hand, restricted sampling can also improve performance: the incorporation of conserved structural constraints into the simulations, as well as the inclusion of the FPP farnesyl analog, significantly improves the identification of farnesylation targets. The performance of different sampling and scoring schemes is summarized in Table S1.

Incorporation of additional FTase backbone conformations from additional FTase-substrate complex structures could enhance the predictions. To examine this, we evaluated the FlexPepBind protocol with two additional backbone templates, and assessed for each the performance on the training set. Using PDBs 1tn7 [9] and 2h6f [57], we achieve comparable and slightly worse AUC values of 0.85 and 0.75, respectively. Combining the scores based on 1tn6 and 1tn7 gave a marginally better performance (AUC = 0.88) and could indeed represent an avenue for future improvement of the protocol.

In addition to sampling, calibration of the energy function can also improve the prediction of binding peptides. In a study on PDZ-peptide interactions, Kaufmann *et al.* optimized the Rosetta energy function on 28 peptide interactions with PDZ domain 3 of PSD-95 for binding prediction. The resulting interface energy using an increased contribution of the hydrogen bond term produces a ROC plot with an AUC value of 0.78 on a general set of 144 peptide-PDZ interactions [58].

In our study we find that scoring with the Rosetta energy provided by the peptide provides the best results for the discrimination of active and non-active peptides. This energy includes the internal peptide energy as well as the interface energy, minus a reference energy term that had been previously introduced to optimize sequence recovery in the design of globular proteins [46]. *De-facto*, removal of this term favors (in decreasing order) C,W,F,H,Y,V,I,A,P and disfavors R,Q,N,E,D,K,S,M,T,G,L. Consequently, without this term, hydrophobic residues will be favored, and performance on the training set improved (probably due to the significant proportion of hydrophobic residues in this set, see Figure 4B). Inferior results are obtained using the Rosetta energy score provided by the interface, as well as the total protein structure. In addition, we would like to note that when using FlexPepDock for sampling, averaging the scores of the best 10 models always gives better results than using merely the top-scoring model (see Table S1 for the performance of different scoring functions).

While the FlexPepDock based protocol gives better results, it is computationally expensive, however, and would impede large-scale characterization (even though it may be the method of choice to make specific decisions once a threshold has been determined from the training set). We found that simple minimization worked well for FTase specificity prediction (and is about 500 times faster than the full FlexPepDock-based protocol). This is due to the restricted nature of the binding - three very strong limitations constrain the peptide backbone orientation. Other systems will probably benefit from increased modeling of backbone flexibility.

In summary, proper calibration of the energy function together with conformational sampling provides efficient structure-based characterization of peptide-protein interactions. It has been estimated that up to 40% of the cellular protein-protein interaction network is mediated by peptide-protein interactions [59]. FlexPepBind is generic in the sense that very little prior knowledge is needed in order to predict the specificity profile for a certain peptide-protein interaction. Given a structural template and a small set of known examples, prediction can be made to identify additional putative targets. We therefore anticipate that this approach can be expanded to a large scale by adapting it to additional peptide-protein interaction motifs in the cellular peptide-protein interaction network.

## Methods

### Detailed description of the protocol

#### Template structure

The complex of human FTase with Rab2a C' peptide was selected as template (PDB: 1tn6 [9]), keeping only the four C' residues of the peptide (CNIQ) and a co-crystallized farnesyl analog ([(3,7,11-trimethyl-dodeca-2,6,10-trienyloxycarbamoyl)-methyl]-phosphonic acid) in place. We also evaluated the use of additional templates, such as 1tn7 [9] and 2h6f [57] (see Discussion).

#### Threading and repacking

Different terminal sequences were threaded onto the peptide backbone and their side chains were packed to find the optimal rotameric configuration (FTase side chains were not allowed to move at this time). Extra rotamers were used both for χ1 and χ2 angles during the rotameric search.

#### Extended FlexPepDock protocol

The prediction protocol using Rosetta FlexPepDock [20] included the creation of 100 models for each of the sequences. Models were scored using the scoring scheme described below, and for each sequence the top-scoring model was chosen as representative.

#### Simple minimization protocol

Instead of FlexPepDock, this simpler protocol applies only minimization over all of the peptide's degrees of freedom (*i.e.* all φ/ψ/ω angles, all of the side-chains χ angles, as well as the rigid-body orientation of the peptide), the FTase interface side chains (Cβ within 8Å of the peptide) and the FPP dihedral angles, using the Davidon-Fletcher-Powell (DFP) minimization algorithm with an absolute tolerance of 0.0001, as implemented in the Rosetta modeling suite [21].

#### Modeling with constraints

Both in the extended FlexPepDock, as well as in the simple minimization protocols, simulations were performed under three constraints that ensure the conservation of observed characteristic structural features in the binding site (Figure 1). The cysteine sulfur atom was forced to stay in its position (the Zn^2+^ ion was not included in the modeling, instead distance constraints to the coordinating residues of FTase were used), and the two structurally conserved hydrogen bonds were enforced as well (*i.e.* the hydrogen bonds between C' carboxylate - FTase Q167α and between a_2_ backbone carbonyl oxygen - FTase R202β ˜Constraints were implemented as harmonic distance functions with a standard deviation of ±0.1Å of the original measured lengths. Constraints with a larger standard deviation (±0.25 Å) performed slightly better (see Table S1).

#### Scoring

The chosen score for discrimination between MTO sequences and non-active sequences consists of the sum of the energy contribution of the 4 peptide residues (as calculated by the Rosetta score12 energy function [22]), but excluding a constant reference energy term (E*ref*) which is fixed per amino acid type and was originally introduced to bias for native protein sequences during fixed backbone sequence design [60].

The scoring schemes that were evaluated in this study include: (1) *Total score* - the regular Rosetta score12 for the entire complex; (2) *Interface score -* the score of the complex less the scores of the peptide and receptor when pulled apart. This score accounts only for interactions across the interface; (3) *Peptide score -* the sum of the energy contribution of the 4 peptide residues; (4) *Peptide score no Ref.* **:** same as Peptide score excluding a constant reference energy term (E*ref*) which is fixed per amino acid type and was originally introduced to bias for native protein sequences, and (5) *iBSA* **:** Buried surface area of the interface. Table S1 summarizes the performance of these different scoring schemes on the training set.

### Genome scan

Human SwissProt [24] was downloaded from IPI [61] (newest version available as of 19.01.10), and was scanned for sequences containing a Cxxx regular motif as the last 4 residues in the protein sequence.

### GO enrichment analysis

Gene Ontology [25] terms were associated with each of the 167 identified candidates for farnesylation (see Results). Enrichment for different cellular compartments, evaluated using DAVID [62], extracted a subset of 93 proteins that are enriched with 18 GO cellular compartment terms, most of them related to the membrane (see Figure S2).

### PrePS

We used the PrePS web-server [7] to obtain sequence-based predictions on our set of 167 selected proteins. For each protein suggested by our protocol to undergo farnesylation, we calculated its prenylation ability using 30 C-terminal residues as input to the server.

### Experimental procedures

Farnesylation screens were performed using radioactivity assays. Different conditions were used to assess the ability of Cxxx sequences to undergo farnesylation under multiple turnover (MTO) and single turnover (STO) conditions, as detailed below. Peptides that do not undergo farnesylation under either of these conditions were defined as NON (see Hougland *et al.* [17] for more details).

#### Steady-state turnover (multiple turnover conditions)

3 µM dansylated-peptide (dns-TKCxxx) was incubated with 1 µM ^3^H-farnesyldiphosphate and 25 nM rat FTase in 50 mM HEPPSO, pH 7.8, 5 mM TCEP, 5 mM MgCl_2_ at 25°C for two hours. The reaction was quenched with 80∶20 isopropanol:acetic acid and run on a silica TLC plate (8∶1∶1 isopropanol:ammonium hydroxide: water). The TLC plates were visualized by autoradiography. Peptides that were observed to be at least 10–20% reacted, as compared to dns-GCVLS, were considered MTO substrates. Using the assumptions that [peptide] < *K*_M_ and that [FPP] is saturating, the lower limit of this assay is approximately 200–400 M^−1^s^−1^, similar to previous work [17].

#### Single turnover

Single turnover assays were carried out the same way as the MTO assays, except that 1 μM FTase, 0.8 μM ^3^H-FPP, and 3 μM dns-TKCxxx peptide were incubated for one hour before the reaction was quenched. Peptides were considered a STO substrate if at least 10 - 20% of the ^3^H-FPP reacted with the peptide after one hour. The range of reactivity of the STO substrates measured in this study is similar to that observed in other studies [17].

## Supporting Information

Figure S1 **Structural basis of the novel CxxE binding motif.** Models of CYLE (green) CYVE (cyan) CYIE (magenta) CFIE (yellow) peptides bound to FTase (orange) are shown. The models suggest that the negatively charged C’ Glutamate residue of the peptide is stabilized by FTase His149 and forms hydrogen bonds with Trp102 and Ser99. Additional potential interactions with water molecules might exist, but are not modeled.
(PNG)

Figure S2 **According to GO cellular compartment annotation, most of our predicted substrates in the human genome are associated with the membrane, suggesting that they indeed might be farnesylation targets**. A GO cellular compartment enrichment analysis conducted with DAVID [62] discovered 18 GO cellular compartment terms enriched in a subset of 93/167 of the predicted substrate proteins. Red columns indicate the –log(*p-value*); Blue diamonds indicate the number of counts for the term in the dataset.
(PNG)

Figure S3 **FlexPepBind identifies 77 novel putative targets undetected by PrePS.** The plot shows the distribution of PrePS predictions on the set of 167 protein sequences that were predicted to undergo farnesylation by FlexPepBind. Almost half of these sequences were not detected by PrePS (in red). The number of + and – symbols indicates the confidence of PrePS in its prediction of a substrate and non-substrate, respectively.
(PNG)

Dataset S1 **The different peptide sequences datasets used for training and testing in this study. A Training set.** 77 MTO and 51 NON peptide sequences. **B Test set 1.** Secondary synthetic library: 29 MTO and 15 NON peptide sequences. **C Test set 2.** 72 Known FTase substrate sequences (from naturally occurring proteins) **D Test set 3.** Ca_1_a_2_L library containing 24 binding and 17 non-binding peptides.
(XLS)

Table S1 **Optimization of the FlexPepBind protocol on the training set: performance of different schemes.** In this table we report the performance of the FlexPepBind protocol over the training set using different sampling and scoring schemes.
(DOCX)
