# Supplementary figures and images for: Identification of a Novel Class of Farnesylation Targets by Structure-Based Modeling of Binding Specificity
Source: PLoS Comput Biol. 2011 Oct 6;7(10):e1002170. doi: 10.1371/journal.pcbi.1002170 (PMC3188499; doi:10.1371/journal.pcbi.1002170)

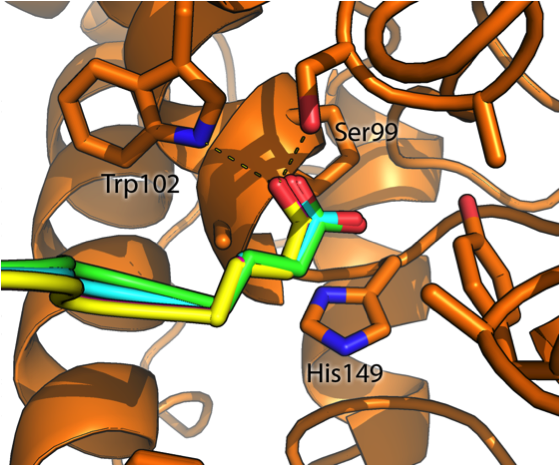

Supplement: Figure S1 — Structural basis of the novel CxxE binding motif. Models of CYLE (green) CYVE (cyan) CYIE (magenta) CFIE (yellow) peptides bound to FTase (orange) are shown. The models suggest that the negatively charged C’ Glutamate residue of the peptide is stabilized by FTase His149 and forms hydrogen bonds with Trp102 and Ser99. Additional potential interactions with water molecules might exist, but are not modeled. (PNG) [file pcbi.1002170.s001.png]

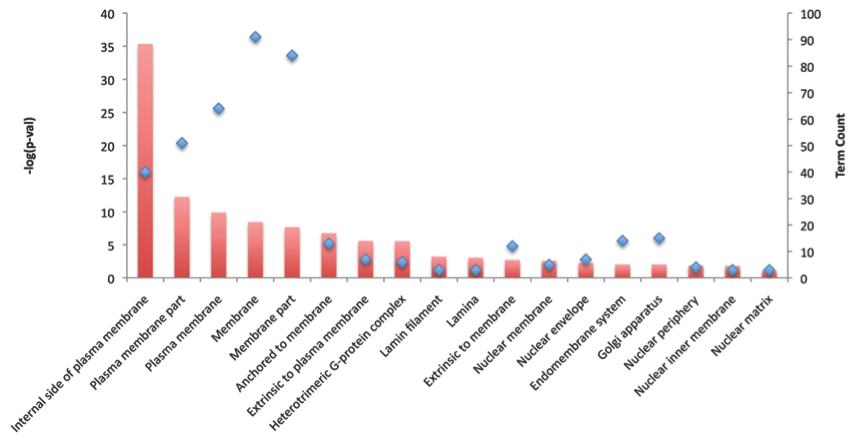

Supplement: Figure S2 — According to GO cellular compartment annotation, most of our predicted substrates in the human genome are associated with the membrane, suggesting that they indeed might be farnesylation targets. A GO cellular compartment enrichment analysis conducted with DAVID [62] discovered 18 GO cellular compartment terms enriched in a subset of 93/167 of the predicted substrate proteins. Red columns indicate the –log(p-value); Blue diamonds indicate the number of counts for the term in the dataset. (PNG) [file pcbi.1002170.s002.png]

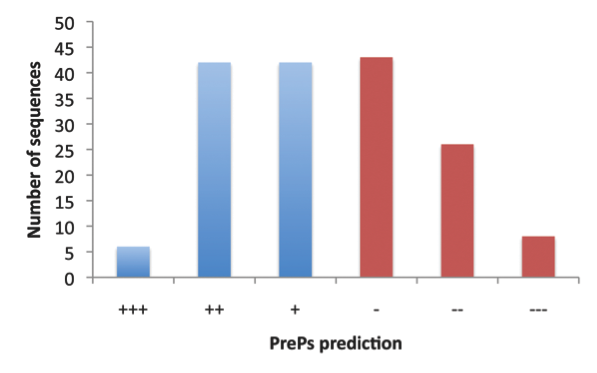

Supplement: Figure S3 — FlexPepBind identifies 77 novel putative targets undetected by PrePS. The plot shows the distribution of PrePS predictions on the set of 167 protein sequences that were predicted to undergo farnesylation by FlexPepBind. Almost half of these sequences were not detected by PrePS (in red). The number of + and – symbols indicates the confidence of PrePS in its prediction of a substrate and non-substrate, respectively. (PNG) [file pcbi.1002170.s003.png]
