# Supplementary material for: Identification of a Novel Class of Farnesylation Targets by Structure-Based Modeling of Binding Specificity
Source: PLoS Comput Biol. 2011 Oct 6;7(10):e1002170. doi: 10.1371/journal.pcbi.1002170 (PMC3188499; doi:10.1371/journal.pcbi.1002170)
Supplement: Table S1 — Optimization of the FlexPepBind protocol on the training set: performance of different schemes. In this table we report the performance of the FlexPepBind protocol over the training set using different sampling and scoring schemes. (DOCX) [file pcbi.1002170.s005.docx]

Table S1. Optimization of the FlexPepBind protocol on the training set: performance of different schemes

| *Sampling^a^* | *FPP^b^* | *Constraints^c^* | *Scoring^d^* | *AUC^e^* |
| --- | --- | --- | --- | --- |
| *Ineffective measures* | | | | |
| FlexPepDock | - | - | iBSA | 0.508 |
| FlexPepDock | + | + | iBSA (Avg. 10) | 0.553 (0.566) |
| *Importance of FPP+constraints* | | | | |
| FlexPepDock | - | - | Total score (Avg. 10) | 0.66 (0.678) |
| FlexPepDock | - | - | Interface score (Avg. 10) | 0.665 (0.704) |
| FlexPepDock | - | - | Peptide score (Avg. 10) | 0.769 (0.804) |
| FlexPepDock | + | + | Interface score (Avg. 10) | 0.798 (0.834) |
| FlexPepDock | + | + | Peptide score (Avg. 10) | 0.836 (0.859) |
| FlexPepDock | + | + | Total score (Avg. 10) | 0.884 (0.887) |
| *Backbone flexibility* | | | | |
| Repacking Ex. Rot. | + | - | Peptide score no Ref. | 0.606 |
| Minimization | + | + | Peptide score | 0.794 |
| Minimization Ex.Rot. | + | + | Peptide score | 0.789 |
| **Minimization** | **+** | **+** | **Peptide score no Ref.** | **0.875** |
| Minimization Ex.Rot. | + | + | Peptide score no Ref. | 0.862 |
| FlexPepDock | + | + | Peptide score no Ref. (Avg 10) | 0.915 (0.933) |
| *Effect of adding constraints* | |  |  |  |
| FlexPepDock | + | - | Peptide score no Ref. (Avg 10) | 0.897 (0.928) |
| FlexPepDock | + | + | Peptide score no Ref. (Avg 10) | 0.915 (0.933) |
| FlexPepDock | + | + (0.25) | Peptide score no Ref. (Avg 10) | 0.936 (0.94) |

| *Dependence on receptor template (PDBid)* | *Peptide sequence* | *AUC^e^* |
| --- | --- | --- |
| **1tn6** | **CNIQ** | **0.875** |
| 1tn7 | CVIF | 0.85 |
| 2h6f | CVLS | 0.75 |
| 1tn6+1tn7 (sum of ranks) |  | 0.88 |

The final scheme used throughout this study is indicated in bold.

^a^ Sampling method as described in the methods section: **Repacking** of the peptide side-chains using rotamer representation, additional **Minimization** all of the peptide degrees of freedom, or using **FlexPepDock** to generate 100 models. **Ex. Rot. –** the rotamer library was supplemented with additional rotamers (using the -ex3 level 7 flag in Rosetta).

^b^ Indicates whether the farnesyl analog FPP is present within the binding pocket in the simulation.

^c^ Indicates whether constraints enforced the Zn^2+^ coordination and two conserved hydrogen bonds during the simulation. Regular constraints were implemented as a harmonic function centered around the distance measured in the native structure with a width of 0.1Å standard deviation. **(0.25)** Constraints are the same, but with a standard deviation of 0.25Å.

^d^ The following scoring schemes were evaluated: **(1) Total score:** regular Rosetta score12 for the entire complex. **(2) Interface score:** score of the complex less the scores of the peptide and receptor when pulled apart; Accounts only for interactions across the interface. **(3) Peptide score:** sum of the energy contribution of the 4 peptide residues. **(4) Peptide score no Ref.:** same as Peptide score excluding a constant reference energy term (E*ref*) which is fixed per amino acid type and was originally introduced to bias for native protein sequences, and **(5) iBSA:** Buried surface area of the interface.

**(Avg. 10)** indicates the average of a given score over the top 10 models produced by FlexPepDock. (**Std. Dev. 10/20)** indicate the standard deviation over the top 10/20 models produced by FlexPepDock.

^e^ The calculated Area Under the Roc (AUC) for this scheme on the training set of this study (Dataset S1A).
